# Loneliness and Social Engagement in Older Adults Based in Lombardy during the COVID-19 Lockdown: The Long-Term Effects of a Course on Social Networking Sites Use

**DOI:** 10.3390/ijerph17217912

**Published:** 2020-10-28

**Authors:** Elena Rolandi, Roberta Vaccaro, Simona Abbondanza, Georgia Casanova, Laura Pettinato, Mauro Colombo, Antonio Guaita

**Affiliations:** 1 Golgi Cenci Foundation, Corso San Martino 10, 20081 Abbiategrasso, Italy; e.rolandi@golgicenci.it (E.R.); r.vaccaro@golgicenci.it (R.V.); s.abbondanza@golgicenci.it (S.A.); l.pettinato@golgicenci.it (L.P.); m.colombo@golgicenci.it (M.C.); a.guaita@golgicenci.it (A.G.); 2 IRCSS-INRCA National Institute of Health & Science on Ageing, Centre for Socio-Economic Research on Ageing, 60124 Ancona, Italy

**Keywords:** social networking sites, lockdown, loneliness, social isolation, communication technology

## Abstract

Older adults are less familiar with communication technology, which became essential to maintain social contacts during the COVID-19 lockdown. The present study aimed at exploring how older adults, previously trained for Social Networking Sites (SNSs) use, experienced the lockdown period. In the first two weeks of May 2020, telephone surveys were conducted with individuals aged 81–85 years and resident in Abbiategrasso (Milan), who previously participated in a study aimed at evaluating the impact of SNSs use on loneliness in old age (ClinicalTrials.gov, NCT04242628). We collected information on SNSs use, self-perceived loneliness, and social engagement with family and friends. Interviewed participants were stratified as trained (N = 60) and untrained (N = 70) for SNSs use, based on their attendance to group courses held the previous year as part of the main experimental study. The groups were comparable for sociodemographics and clinical features. Participants trained for SNSs use reported significantly higher usage of SNSs and reduced feeling of being left out. Compared to pre-lockdown levels, individuals trained for SNSs use showed a lighter reduction in social contacts. These findings support the utility of training older adults for SNSs use in order to improve their social inclusion, even in extreme conditions of self-isolation and perceived vulnerability.

## 1. Introduction

The COVID-19 outbreak caused unprecedented health, social, and economic challenges worldwide. The first COVID-19 Italian case was detected in Lombardy on 21 February 2020 and soon after, Italy became the first country for number of infections besides China [1]. Older people are at greater risk of infection and mortality from COVID-19—in Italy, respectively, 61 and 82 years of median age, while Lombardy residents represented 49.1% of the deceased [2]. Therefore, being more than eighty years old and being a resident of Lombardy is the most risky profile for both infection and mortality. The Italian government established a nationwide lockdown on March 9th, allowing only essential transfers for grocery, health, or work [3]. Although necessary to counteract the spread of the COVID-19 epidemic, the strict self-isolation measurements may disproportionally affect older adults’ psychological and social wellbeing [4,5]. Indeed, loneliness feelings and social isolation were already seen as a public health issue for older adults, with deleterious impact on physical, cognitive, and mental health [5,6,7]. Older adults are also less familiar with information and communication technology (ICT) and social networking sites (SNSs) use [8,9,10], which became essential to maintain social contacts during the quarantine.

The aim of this study is to explore whether being trained for SNSs use had a significant impact on SNSs use, loneliness feelings, and social isolation during the COVID-19 lockdown period, in community-dwelling old-old adults (81–85 years) living in Abbiategrasso, a city in the Milan metropolitan area (Lombardy region). We conducted a telephone survey on participants of “The Ageing in a Networked Society-Social Experiment Study (ANS-SE)”, a randomized controlled trial aimed at exploring the causal impact of SNSs use on loneliness and social isolation in old age [11]. The ANS-SE study enrolled older adults without previous experience in SNSs use to be randomly allocated to one of three conditions: a course on SNSs use (experimental group), waiting list (inactive control group), and lifestyle education course (active control group). For the purposes of the present investigation, survey respondents were stratified as trained for SNSs use if they attended the course on SNSs use before the COVID-19 outbreak, thus including both participants randomized to the experimental group and those randomized to the waiting list who subsequently accepted to attend the course after the experimental phase (June 2019). Therefore, adopting a quasi-experimental design, we were able to test both cross-sectional and longitudinal differences between older adults trained and untrained for SNSs use during the COVID-19 lockdown. Indeed, the outcome measures collected during the experimental phase (pre–post intervention) were used as a proxy of pre-lockdown levels of loneliness and social isolation of the survey participants. With regard to the ANS-SE study experimental phase, no statistically significant effects of SNSs course on loneliness and social isolation were found in the short term (unpublished work).

Loneliness is the negative feeling derived from the discrepancy between the desired and the actual personal social network, while social isolation usually refers to the objective characteristic of the social network (i.e., number of kin and non-kin relationships) [12]. Observational studies on oldest-old individuals suggest a positive association between ICT use and different aspects of subjective well-being, including loneliness [13,14]. However, a recent review from our group showed that very few studies tested this association using experimental or quasi-experimental design, which is necessary to draw conclusions on the causal relationship between variables [15]. Systematic reviews of interventional studies show that training on ICT use had beneficial short-term effects on social connectedness, social support, and social isolation in older adults [16,17]. The results on loneliness are mixed, with the majority of studies reporting beneficial effects, while some report none or negative effects [16,17]. Few interventional studies performed long-term follow-up, and the results available on the maintenance of the gain observed post training are not conclusive [16]. Moreover, studies in this research area showed several limitations, which prevent us from drawing firm conclusions on the causal effect of ICT and SNSs use on loneliness and social isolation, such as: small and/or convenience samples, lack of active control group and great heterogeneity in the outcome measures used [15,16,17,18,19]. In this context, the ANS-SE study design has several methodological strengths: sample size based on power analysis, outcome measures selected to assess both loneliness and social isolation with internationally validated scales, randomization of participants, and the presence of active and inactive control conditions [11]. Furthermore, a one-year follow-up was planned to provide evidence on the long-term effects of the course on SNSs use.

However, the COVID-19 pandemic dramatically changed the psychological and social context worldwide, as well as the possible modalities for data collection. At the same time, the topic of social isolation and loneliness among older adults has acquired even more relevance. Feeling lonely as a result of the pandemic was the most common personal stressor identified in an Australian survey [20]. Older adults have received stricter directives on social distancing, as they were one of the first groups encouraged to stay home worldwide [21]. Several commentaries proposed ICT use improvement in older populations as a potential strategy to counteract social isolation and loneliness during the pandemic [4,5,20,21,22,23,24]. Keeping connections is listed among the tips for preventing the detrimental effect of loneliness and social isolation [5]. The use of digital technologies can bridge social distance, even while physical distancing measures are in place [24]. Many older adults, however, might not be familiar with these new technologies [5,8,10]. However, training on SNSs use may prove useful to familiarize older adults with such technologies [22].

Here, we had the unique opportunity to investigate empirically the long-lasting effect of training on SNSs use in oldest-old adults on SNSs use, loneliness, and social isolation, in the context of the COVID-19 quarantine. To this end, a telephone survey was conducted soon after the first attenuation of the Italian lockdown, when visiting family members was allowed after nearly 2 months of self-isolation. Based on the available evidence and on the aforementioned expert opinion in the field, we hypothesized that being trained for SNSs use, within a context in which any in-person social contact outside home is forbidden, could potentially help older adults in maintaining pre-lockdown levels of social engagement, with a potential benefit on perceived loneliness during the quarantine period. To the best of our knowledge, our study provides the first empirical evidence on the effectiveness of training community-dwelling oldest-old adults on SNSs use to counteract social isolation during the quarantine.

## 2. Materials and Methods

### 2.1. Participants and Procedures

The study protocol of the ANS-SE study is described in detail elsewhere [11]. Briefly, participants were selected among older adults participating in the fourth evaluation wave of the InveCe.Ab study (Invecchiamento Cerebrale in Abbiategrasso), a population-based cohort set in Abbiategrasso, a town near Milan [25]. The ANS-SE study eligibility criteria were: (i) consent to be contacted to take part in further studies; (ii) a score lower than 9 on the 15-item Geriatric Depression Scale (GDS); (iii) absence of functional limitations to hands and visual or hearing impairment, which might prevent smartphone use; (iv) any diagnosis of dementia or mild cognitive impairment (MCI) and a Mini-Mental State Examination score higher than 24; (v) no experience in using SNSs. The setting and location of the ANS-SE study was the Golgi Cenci Foundation in Abbiategrasso. 

The sample size calculation is reported elsewhere [11]. Briefly, power analysis was conducted using an estimated effect size of 0.25 with 80% power using a 5%-level F test of the equality of means, assuming a balanced design. According to statistical computing, a sample size of no less than 52 subjects per group was required. Thereafter, a 10% attrition rate was considered, and the expected sample size resulted in no less than 57 subjects per group.

The recruitment phase was conducted on January 2019: 180 InveCe.Ab participants presenting the ANS-SE study eligibility criteria were randomly allocated to one of three conditions: a course on SNSs use (experimental group), waiting list as an inactive control group, and lifestyle education course as an active control condition. They were contacted for the study proposal and 144 participants underwent baseline evaluation—they represent the sample for this investigation.

Group interventions took place between 18 February and 7 March 2019. The SNSs course consists of 5 interactive group sessions held twice a week. The themes covered were: smartphone use, Facebook and WhatsApp use, privacy rules, and fraud risk prevention using Facebook. Participants were provided with user-friendly smartphones designed for older adults. For the remaining 3 weeks of the experimental phase, two trainers were available twice a week for face-to-face tutoring. Moreover, they regularly sent messages and media in a dedicated WhatsApp group and Facebook pages.

Participants underwent pre–post evaluations in order to verify the short-term effect of the intervention on loneliness and social isolation (primary outcomes), as well as on executive functions (secondary outcomes). Furthermore, to explore the long-term effects of the intervention, a one-year in-person follow-up was planned. However, due to the strict lockdown measurements deployed in the Lombardy region from 9 March 2020 to counteract the spread of the COVID-19 epidemic, we performed telephone surveys to explore how participants in the ANS-SE study were experiencing the lockdown period. 

Participants were contacted between 4 May and 19 May 2020, one year after post-intervention evaluations for the ANS-SE study (May 2019) and soon after the first attenuation of the lockdown measurements. Indeed, the so-called phase 2 of the Italian lockdown started on 4 May when visiting family members was allowed after nearly 2 months of self-isolation. During the phone call, we collected information on SNSs use, self-perceived loneliness measured by the 3-item UCLA (University of California, Los Angeles) loneliness scale, and social engagement measured by the 6-item Lubben Social Network Scale (LSNS-6). We explicitly asked participants to answer by referring to the phase 1 lockdown that had just ended.

We were thus able to test both cross-sectional and longitudinal differences between older adults trained and untrained for SNSs use during the COVID-19 lockdown. Cross-sectional differences were investigated by comparing the two groups on measures of SNSs use, loneliness, and social engagement collected during the telephone survey. Longitudinal differences were investigated by comparing one-year changes in levels of loneliness and social engagement, considering the average of corresponding measures collected before and after the ANS-SE experiment as a proxy of pre-lockdown status.

Among the 144 eligible ANS-SE study participants, 1 died, 4 refused, and 9 were unreachable, resulting in a sample of 130 survey participants. For the purposes of the present investigation, participants were stratified as trained (N = 60) and untrained (N = 70) for SNSs use. Survey respondents were stratified as trained for SNSs use if they attended the course before the COVID-19 outbreak, thus including both participants randomized to the experimental group and those randomized to the waiting list who attended the course in June 2019, at the end of the experimental phase (N = 15).

The ANS-SE study received approval from the Ethics Committee of the University of Milano Bicocca (prot. 431/2019). The study was performed in accordance with the guidelines of the Declaration of Helsinki. All participants signed the written informed consent at the beginning of the ANS-SE study.

### 2.2. Measures

SNSs use. Participants were asked about their use of Facebook and WhatsApp during the phase 1 lockdown (yes/no response). Furthermore, usage frequency was reported with a 4-point scale: 1 = less than once a week; 2 = once a week; 3 = several times per week; 4 = daily.

Loneliness. Self-perceived loneliness was evaluated through the administration of the 3-item UCLA loneliness scale, an abbreviated tool specifically developed for telephone surveys addressed to older adults [26]. The scale consists of the following items: “How often do you feel that you lack companionship?”; “How often do you feel left out?”; “How often do you feel isolated from others?” Participants rate each item on a 3-point scale: 1 = hardly ever; 2 = sometimes; 3 = often. The total score consists of the sum of the items, ranging from 3 to 9. This score was used as the loneliness measure in our analysis. We further reported the response to each item dichotomized (i.e., absence = hardly ever; presence = sometimes or often), to show cross-sectionally the distribution of specific loneliness feelings according to group. 

In the ANS-SE study, we administered in person the Revised UCLA loneliness scale before and after the end of the experimental phase, which consists of 20 items on a 4-point Likert scale (1 = never; 2 = rarely; 3 = sometimes; 4 = often) [27]. However, the instrument is not suitable for telephone surveys, while the 3-item UCLA loneliness scale was specifically designed for this purpose and showed high correspondence with the same 3 items when asked in the full in-person scale [26]. Thus, to obtain the baseline level for the longitudinal analysis, the relevant 3 items asked in the full scale were re-conducted to a 3-point Likert scale by recoding both the “never” and “rarely” response to the “hardly ever” response of the 3-item version. The items were then summed up to obtain the total score. Finally, the pre–post scores were averaged to obtain a unique pre-lockdown level of loneliness, both for total score and for individual items.

Social engagement. Social engagement with family and friends was explored with the 6-item Lubben Social Network Scale (LSNS-6, [28]). The LSNS-6 is composed of a set of 3 questions to evaluate their relationship with relatives, followed by the same set of questions referring to friends. Specifically, the questions were “How many relatives/friends do you see or hear from at least once a month?”; “How many relatives/friends do you feel close to such that you would call on them for help?”; “How many relatives/friends do you feel at ease with that you can talk about private matters?” The items were thus suitable to report both remote and in-person contacts and to capture both structural and functional aspects of the social network [29]. Items were rated on a 6-point Likert scale: 0 = none; 1 = one; 2 = two; 3 = three or four; 4 = five through eight; 5 = nine or more. The total score was the sum of the 6 items, ranging from 0 to 30 (LSNS-6 Total), with higher scores indicating higher engagement (i.e., lower social isolation). Moreover, subscales were obtained by adding the items on relatives (LSNS-6 Family, range 0–15) and friends (LSNS-6 Friends, range 0–15). The same scale was administered during the ANS-SE study experimental phase. Pre-lockdown levels of social engagement were obtained by averaging the pre–post score of the LSNS-6 scale and subscales. 

### 2.3. Statistical Analysis

Statistical analysis was performed on Statistical Package for Social Science (IBM Corp. Released 2011. IBM SPSS Statistics for Windows, Version 20.0. Armonk, NY, USA: IBM Corp.). To ascertain whether the groups were comparable for relevant sociodemographic and clinical features, an Independent Sample T-test for continuous variables (age, education, GDS, MMSE, comorbidity) and a Chi-square test for categorical variables (sex, percentage of living alone) were performed. 

First, we tested cross-sectionally whether being trained for SNSs use before the COVID-19 outbreak (independent variable) was associated with the outcomes of interest collected during the telephone survey (dependent variables). More specifically, we investigated between groups (trained vs. untrained) differences in SNSs use (yes/no), loneliness (UCLA total score and individual items dichotomized), and social engagement (LSNS-6 scale and subscales), using a Chi-square test for dichotomous variables and Mann–Whitney test for continuous variables (skewed distribution). Furthermore, Spearman’s rank-order correlations were performed to explore any direct or inverse relationship between SNSs usage frequency and measures of loneliness and social engagement. 

We then performed longitudinal analyses to investigate the effect of SNSs use training on one-year changes in loneliness and social isolation. As previously detailed, proxies of pre-lockdown levels of loneliness and social engagement were obtained retrospectively by averaging the corresponding measures collected before (February 2019) and after (May 2019) the experimental phase of the ANS-SE study. A delta score was computed for each measure (post-lockdown–pre-lockdown). Thus, one-way analysis of covariance (ANCOVA) was used to test the effects of being trained on SNSs use on social engagement changes (LSNS-6 scale and subscales) after the COVID-19 lockdown, including the pre-lockdown measure as a covariate. Generalized Linear Models with identity link functions were performed for changes in UCLA scale and individual items, including the pre-lockdown measure as a covariate, since they displayed a skewed distribution. 

## 3. Results

Descriptive characteristics of the study sample are reported in Table 1. Older adults trained and untrained for SNSs use were comparable for key sociodemographics and clinical features, as well as for the percentage of individuals living alone during the lockdown period (Table 1).

Table 2 shows the main findings of the present study. The first step of this study is to explore cross-sectional differences between trained and untrained participants on SNSs use, loneliness, and social engagement during the COVID-19 lockdown. The findings underline how participants trained for SNSs use reported significantly higher usage (yes/no response) of Facebook (χ^2^(1) = 17.1, *p* < 0.001) and WhatsApp (χ^2^(1) = 11.92, *p* = 0.001). However, we must observe that a portion of participants in the untrained group reported being SNSs users during the lockdown period: 31% WhatsApp users; 7% Facebook users (Table 2). As regards loneliness and social engagement, non-significant differences were found for the UCLA loneliness scale total score and the LSNS-6 scores. However, considering the prevalence of yes responses to the individual items of the UCLA loneliness scale, a lower portion of trained participants reported a feeling of being left out (χ^2^(1) = 5.11, *p* = 0.024) and a trend of reduced feeling of isolation was detected (χ^2^(1) = 3.60, *p* = 0.058).

When longitudinal analyses were performed to test the impact of SNSs training on one-year change in the measures of interest, a slight reduction in social engagement was found in the trained group, on both LSNS-6 Total (F(1) = 7.85, *p* = 0.006) and LSNS-6 Family (F(1) = 8.16, *p* = 0.005). No significant differences between groups were found for the UCLA scale and single items using a generalized linear model.

Finally, Table 3 showed an exploratory analysis on the eventual inverse or direct relationship between SNSs usage frequency and loneliness feelings (UCLA individual items) or social engagement (LNSNS-6 scale and subscale). No significant associations were discerned. Only a trend toward significance was found for an inverse relationship between WhatsApp usage frequency and feeling of a lack of companionship (ρs(56) = −0.257, *p* = 0.051; Table 3).

## 4. Discussion

The aim of the present study was to investigate whether older adults trained for SNSs use showed more frequent use of SNSs and increased resilience (i.e., reduced loneliness and social isolation) during the period of forced self-isolation due to the COVID-19 outbreak. The main findings of the study are:

(1) SNSs use was higher in the trained group;
(2) The feeling of being left out was less frequent in the trained group during the lockdown;
(3) Participants trained for SNSs use showed a lighter reduction in social engagement compared to the pre-lockdown level.

### 4.1. Relevance of Results 

These results support a better understanding of SNSs use by older people. In particular, the findings confirm the efficacy of training to use SNSs for older adults and their positive effect on increasing their perceived social inclusion and maintaining meaningful social contacts and relationships, even in adverse and threatening conditions. Both aspects are relevant for several reasons. Firstly, widespread SNSs use by elders reduces the grey digital divide, which is the difference in ICT access and use between the aged population and younger generations. Indeed, although the portion of ICT users within the aged population has constantly increased in recent years, the grey digital divide is still an issue in many countries [9,30,31]. The relevance of this issue grows for countries like Italy, where the percentage of ICT and SNSs users among older adults is one of the lowest in Europe [8,10].

Our findings clearly show the advantage of formal training in this accelerated process of digitalization. Even a short course delivered in groups has the potential to promote long-term SNSs use in older adults lacking previous experience with social media and, in most cases, with the smartphone itself. This assumption finds confirmation in the significantly higher proportion of SNSs users within the trained group one year after SNSs course completion. This is not a trivial finding, since no further tutoring was provided after the end of the experimental phase. 

Moreover, the present study provides evidence of the effectiveness and appropriateness of training for SNSs use in older adults aged 80 years and over (oldest-old). All these assumptions work to contrast the ageism stigma on communication technologies use [32]. The cross-sectional comparison shows no significant differences between trained and untrained participants on scales measuring loneliness and social isolation as a whole. However, between-group differences emerged when the individual items of the UCLA loneliness scale were considered. These results highlight the benefit of SNSs use on specific loneliness feelings. Moreover, these detected benefits remain quite stable in the middle term. In particular, after one year, having been trained had a beneficial impact on one-year change in social engagement, especially with family. This finding underlines the strong linkage between loneliness feelings and social participation [33,34].

As for the SNS used, the older adults in this study showed a marked preference for WhatsApp over Facebook usage. This could be due to the specific features of the SNSs considered. Indeed, while WhatsApp is mainly used to maintain social contact with one’s current social network, Facebook requires the construction of a public or semipublic profile and an extended social network with which to share content, and it might thus raise privacy concerns. Systematic reviews aimed to explore the experience of older SNSs users showed as a main benefit the possibility to maintain social contact with family and friends and to enter an intergenerational communication with younger family members, while several barriers were reported, such as privacy concerns, technical difficulties, and low accessibility/usability [17,35,36].

### 4.2. Contributions for the Literature 

This study supports the literature debate on older adults’ loneliness and social isolation and on the strategies to contrast these negative effects on their wellbeing. Several commentaries on older adult needs during the COVID-19 pandemic highlighted social inclusion and loneliness reduction among public health priorities and proposed improved ICT use as a potential strategy [4,5,20,21,22,23,24].

Firstly, the study confirms the multidimensional characteristics of loneliness and social isolation definitions, underlining their interrelated constructs. Indeed, in order to gain a deep understanding of these phenomena, multidimensional measures must be applied, covering both structural and functional aspects of the social network, as well as subjective feelings on social relationships [29]. The lack of significance of SNSs use on changes in loneliness feelings is in line with the literature, which showed contrasting results [16,37]. Loneliness is conceptualized as a quite stable trait, with high one-year test–retest reliability [26], and a recent meta-analysis on longitudinal studies shows great stability of loneliness from adolescence to old age [38]. This could potentially explain the inconsistency of findings on loneliness outcomes in interventional studies so far. However, it is possible that more sensitive and broad measures of loneliness, not suitable for phone interviews, would help us fully grasp subtle oscillations in a rather stable tract. 

Moreover, while we were conducting the present investigation, other research articles were published on the effects of self-isolation due to COVID-19 on older adults’ well-being and loneliness, further confirming the relevance of the topic. Online surveys conducted in Spain and Israel showed that a negative self-perception of aging was associated with loneliness and psychological distress [39,40]. Interestingly, the study conducted in Spain, including 1310 participants aged between 18 and 88 years, showed an inverse association between chronological age and both loneliness and distress, suggesting better coping abilities in aged individuals [39]. A longitudinal observational study showed that US older adults experienced increased depressive symptoms and loneliness than prior to the COVID-19 pandemic [41]. Conversely, in a large Swedish cohort evaluated annually for 5 years, no changes in loneliness were detected [42].

This study lends its contribution to the specific literature focusing on these issues, enlarging the international perspective. Our results, together with the incoming literature, shed light on the resilience and personal resources of the older population facing the adversities of the COVID-19 outbreak. This is an important message, since aging is often described only in terms of vulnerabilities and losses.

Lastly, our study contributes to promoting further experimental studies on ICT or SNSs use, showing the effects on loneliness and social isolation over a long-term observation time. Few previous studies on the topic performed long-term follow-up, which is generally scheduled no more than 6 months after the end of the training phase [16].

### 4.3. Strengths and Limitations of Study

Our study had several strengths and novelties. The longitudinal measurements allow the detection of changes before and after the pandemic. The quasi-experimental design allows for a robust interpretation of the results, suggesting a cause–effect mechanism between the course on SNSs use and increased maintenance of social networks during the pandemic. Moreover, very few studies evaluated the long-term effects of this kind of intervention. By including participants aged 80 years and over, we reported data on a scarcely studied population (oldest-old, old-old) which, however, represent a relevant and growing portion of the general one. Actually, recent observational studies suggested a positive association between ICT use and well-being even among the oldest-old [13,14]. Our study experimentally showed the possibility of promoting long-term SNSs use in oldest-old individuals without previous experience with it. Another relevant novelty is the use of smartphone devices, which was uncommon among interventional studies in the field [16,17]. 

Some limitations should also be discussed. As mentioned earlier, some outcomes would be better characterized by in-person evaluation (i.e., loneliness). The results presented are not widely generalizable, as we selected participants without cognitive impairment or clinically relevant depressive symptoms. It is, therefore, possible that we selected the most resilient individuals, in terms of both cognitive and psychological health. Moreover, even if SNSs use was significantly increased, a non-negligible portion of trained participants did not use it at all. Future studies, including a larger sample to perform stratified analysis, should investigate which personal, motivational, or technical aspects hamper ICT and SNSs use among older adults.

## 5. Conclusions

The present study showed that a short training course on SNSs use has the potential to promote long-term effects in older adults aged 80 years and over. Participants trained for SNSs use showed benefits in specific loneliness feelings and a lighter reduction in social engagement with family and friends during the period of forced self-isolation to counteract the COVID-19 spread, one year after training completion. These findings support the usefulness of training older adults for SNSs in order to improve their social inclusion, even in extreme conditions of self-isolation and perceived vulnerability. However, more studies are still needed to clarify the psychological and social effects of the COVID-19 pandemic in individuals already struggling with mental health or in disadvantaged socioeconomic conditions. Furthermore, since the epidemic involved almost the entire world, it would be of great interest to investigate similarities and differences across countries, in relation with government decisions, media communication styles, and cultural aspects.

## Figures and Tables

**Table 1:** Descriptive characteristics of the study population (N = 130) and comparisons between older adults trained and untrained for Social Networking Sites (SNSs) use.

Characteristics		Study Sample N = 130	Trained for SNSs Use N = 60	Untrained for SNSs Use N = 70	*p* *
Sociodemographics					
Age		81.8 ± 1.4	82.0 ± 1.6	81.6 ± 1.2	0.102
Sex, female		68 (52%)	32 (53%)	36 (51%)	0.828
Education		8.7 ± 3.4	8.7 ± 3.3	8.6 ± 3.5	0.883
Living alone		48 (37%)	23 (38%)	25 (36%)	0.758
Clinical scales	Range				
Comorbidity (CIRS)	14–70	1.7 ± 0.3	1.7 ± 0.3	1.7 ± 0.3	0.438
Depressive symptoms (GDS)	0–15	1.9 ± 1.9	2.0 ± 1.9	1.8 ± 2.0	0.579
Global cognition (MMSE)	0–30	28.4 ± 1.5	28.3 ± 1.4	28.4 ± 1.5	0.503

Values denote mean ± SD for continuous variables, counts (%) for dichotomous variables. * *p* values indicate significance in the Independent Sample *t*-test for continuous variables and Chi-square test for dichotomous variables. Abbreviations: CIRS—Cumulative Illness Rating Scale; GDS—Geriatric Depression Scale (15 item); MMSE—Mini Mental State Examination; SNSs—Social Networking Sites.

**Table 2:** Differences between older adults trained and untrained for Social Networking Sites (SNSs) use on SNSs use, loneliness, and social engagement during the COVID-19 lockdown and impact of SNSs training course on one-year changes in loneliness and social engagement.

Outcomes	Trained for SNSs Use N = 60	Untrained for SNSs Use N = 70	*p*
Cross-sectional analysis *			
SNSs use			
Facebook users	22 (37%)	5 (7%)	**<0.001**
WhatsApp users	37 (62%)	22 (31%)	**0.001**
Loneliness			
UCLA 3-item total	4.2 ± 1.5	4.6 ± 1.6	0.124
Feeling a lack of companionship	31 (53%)	42 (60%)	0.395
Feeling left out	6 (10%)	18 (26%)	**0.024**
Feeling isolated from others	10 (17%)	22 (31%)	0.058
Social engagement			
LSNS-6 Total	14.8 ± 5.6	13.3 ± 4.9	0.102
LSNS-6 Family	8.8 ± 2.9	7.9 ± 2.4	0.063
LSNS-6 Friends	6.0 ± 4.1	5.4 ± 3.2	0.389
Longitudinal analysis ^&^			
Loneliness			
UCLA 3-item total score	0.0 (0.2)	0.3 (0.2)	0.135
Feeling a lack of companionship	0.3 (0.1)	0.3 (0.1)	0.664
Feeling left out	−0.3 (0.1)	−0.1 (0.1)	0.133
Feeling isolated from others	0.2 (0.1)	0.0 (0.1)	0.082
Social engagement			
LSNS-6 Total	−1.7 (0.6)	−3.8 (0.5)	**0.006**
LSNS-6 Family	−0.8 (0.3)	−1.9 (0.3)	**0.005**
LSNS-6 Friends	−1.0 (0.4)	−1.9 (0.4)	0.100

* Cross-sectional analysis: Values denote mean ± SD for continuous variables, counts (%) for dichotomous variables. *p* values indicate significance in Mann–Whitney test for continuous variables, Chi-squared test for dichotomous variables. ^&^ Longitudinal analysis: Values denote adjusted mean (SE). *p* values indicate significance in the one-way ANCOVA (social engagement) or generalized linear model (loneliness) with baseline score as covariate. In bold statistically significant *p* value (*p* < 0.05). Abbreviations: LSNS-6—Lubben Social Network Scale 6-items; SNSs—Social Networking Sites.

**Table 3:** Correlations between Social Networking Sites (SNSs) usage frequency and measures of loneliness and social engagement.

SNSs Usage Frequency		Loneliness			Social Engagement		
		UCLA Lack of Companionship	UCLA Left Out	UCLA Isolated	LSNS-6 Total	LSNS-6 Family	LSNS-6 Friend
WhatsApp usage frequency N = 58	ρs	−0.257	−0.051	−0.018	0.021	0.024	0.009
	*p*	0.051	0.706	0.895	0.875	0.858	0.948
Facebook usage frequency N = 26	ρs	0.286	−0.125	0.102	0.275	0.229	0.227
	*p*	0.157	0.544	0.619	0.174	0.260	0.264

ρs—Spearman’s correlation coefficient. *p* values denote significance at Spearman’s rank-order correlation.
